# Development of vaccine for dyslipidemia targeted to a proprotein convertase subtilisin/kexin type 9 (PCSK9) epitope in mice

**DOI:** 10.1371/journal.pone.0191895

**Published:** 2018-02-13

**Authors:** Ryo Kawakami, Yoichi Nozato, Hironori Nakagami, Yuka Ikeda, Munehisa Shimamura, Shota Yoshida, Jiao Sun, Tomohiro Kawano, Yoichi Takami, Takahisa Noma, Hiromi Rakugi, Tetsuo Minamino, Ryuichi Morishita

**Affiliations:** 1 Department of Cardiorenal and Cerebrovascular Medicine, Faculty of Medicine, Kagawa University, Kagawa, Japan; 2 Department of Clinical Gene Therapy, Osaka University Graduate School of Medicine, Suita, Osaka, Japan; 3 Department of Geriatric and General Medicine, Osaka University Graduate School of Medicine, Suita, Osaka, Japan; 4 Department of Health Development and Medicine, Osaka University Graduate School of Medicine, Suita, Osaka, Japan; 5 Department of Neurology, Osaka University Graduate School of Medicine, Suita, Osaka, Japan; Centro Cardiologico Monzino, ITALY

## Abstract

Proprotein convertase subtilisin/kexin type 9 (PCSK9) regulates expression of low-density lipoprotein (LDL) receptors via receptor internalization and subsequent lysosomal degradation. Thus, an anti-PCSK9 antibody is well known as an anti-hyperlipidemia drug. Here, we aimed to develop vaccine for a long-term treatment of dyslipidemia targeted to PCSK9. In This study, we designed a peptide vaccine for mouse PCSK-9, which consisted of short peptides conjugated to keyhole limpet hemocyanin (KLH) as a carrier protein. Vaccines were administered to male *apolipoprotein E (ApoE) deficient mice* with adjuvants and significantly elicited an antibody response against PCSK9. The PCSK9 vaccines were administered to mice three times in 2-week intervals, and antibody titers and lipoprotein levels were evaluated up to 24 weeks after the first immunization to determine the therapeutic effect. Anti-PCSK9 antibody titers reached peak levels 6 weeks after the first immunization, and theses titers were maintained for up to 24 weeks. Decreased plasma levels of total cholesterol, very low-density lipoprotein (VLDL), and chylomicron (CM) were maintained for up to 24 weeks. Immunized mice exhibited a significant increase in cell-surface LDL receptor expression. Stimulation with KLH, but not PCSK9, induced the production of INF-gamma and interleukin-4 (IL-4), as determined with ELISPOT assays, thus indicating that PCSK9 vaccine did not elicit T-cell activation in our vaccine system. The present anti-PCSK9 vaccine induced long-lasting anti-PCSK9 antibody production and improved lipoprotein profiles. Thus, anti-PCSK9 vaccine could become a new option for the treatment of dyslipidemia as a long-acting therapy in future.

## Introduction

Patients may remain at increased risk of an atherosclerotic cardiovascular disease event despite maximally tolerated statin therapy. The crucial role of proprotein convertase subtilisin/kexin type 9 (PCSK9) in the metabolism of low-density lipoprotein (LDL) and the LDL receptor (LDLR) and the verified safety of PCSK9 inhibition has led to the development of PCSK9 inhibitors [1]. PCSK9 inhibitors, including monoclonal antibodies (mAbs), evolocumab and alirocumab, markedly decrease plasma LDL cholesterol. These mAbs are the most potent cholesterol-lowering agents available and can decrease LDL cholesterol up to 73% in patients with high LDL cholesterol on maximally tolerated statin therapy and ezetimibe, including adult patients with heterozygous or homozygous familial hypercholesterolemia (FH) or statin-resistant patients with an atherosclerotic cardiovascular disease [2, 3]. However, the cost-effectiveness of PCSK9 mAbs is lower despite the efficacy of these agents. A recent analysis has reported that the use of PCSK9 inhibitors for heterozygous FH and atherosclerotic cardiovascular disease costs more than $14,000 per patient per year, and this treatment would not be cost effective unless the annual costs were decreased to $4536 (the threshold to meet $100,000 per quality-adjusted life-year) [4]. This expense highlights the need for less expensive PCSK9 inhibitors for use as alternative or adjunct agents to PCSK9 mAbs to decrease the required dose and cost of treatment. PCSK9 mAbs are administered via biweekly or monthly subcutaneous injection, thus introducing practical and logistic barriers to compliance, as compared with daily oral medications, such as statins. A therapeutic vaccine therapy that could produce a sustained presence of functional antibodies would decrease total protein profile and provide an alternative approach using long-acting agents to effectively target anti-PCSK9 and prevent and treat atherosclerotic cardiovascular disease in the future.

We developed an anti-PCSK9 peptide vaccine that increased anti-PCSK9 antibody titer for up to 24 weeks. Three injections of this vaccine at 2-week intervals increased the cell-surface expression of the LDLR in liver and improved plasma lipoprotein profiles in male *apolipoprotein E (ApoE)-deficient mice*. We selected short (9–10 amino acids) peptides as antigens, which may not directly bind major histocompatibility complex (MHC) class 2 molecules and hence may avoid an auto-immune response while specifically inducing anti-PCSK9 antibody production.

## Materials and methods

### Vaccine design and synthesis

Two antigen peptides were selected to generate neutralizing antibodies against PCSK9 area V1, which spans a site in the C-terminal sequence of PCSK9 (580–589 amino acids), and V2, which spans the 682–690 amino acid sequence. The N-terminus of each candidate peptide was conjugated to KLH via glutaraldehyde, and the synthetic peptides were purified with reverse-phase high-performance liquid chromatography (>99% purity) (PEPTIDE INSTITUTE Inc., Osaka, Japan) as described previously [5].

### Animals

This study was carried out in strict accordance with the recommendations in the Guide for the Care and Use of Laboratory Animals of the National Institutes of Health. The protocol was approved by the Ethical Committee for Animal Experiments of the Osaka University Graduate School of Medicine (27-020-024). Eleven-week-old male *ApoE deficient mice* were provided from KAC Co., Ltd., housed in a temperature- and light cycle-controlled facility, and given free access to food and water. *ApoE-deficient mice* with a thymine (T) insertion mutation in exon 4 were generated with the CRISPR/Cas9 system [6]. This out-of-frame mutation caused the appearance of a stop codon at the 83rd codon from the insertion site (S1 Fig).

### Treatment and immunization schedule

Peptide solutions were mixed in an equal volume of complete or incomplete Freund’s adjuvant (Wako Pure Chemical Industries, Ltd., Osaka, Japan) before immunization. Animals were anesthetized with inhaled isoflurane (4% for induction, 2% for maintenance). Animals were immunized subcutaneously (s.c.) with each vaccine (V1 or V2) or each dose (5 μg or 50 μg) of peptide antigen (N = 4–7). Control groups were injected with an equal quantity of KLH or saline mixed with an equal volume of Freund’s adjuvant (N = 4). A 100-μL blood sample was collected from the tail vein before each immunization and transferred to heparin-containing tubes. All plasma samples were stored at −80°C for further analysis. Two or three immunizations were performed in biweekly intervals, and short-term experiments were finalized 2 weeks after the last immunization (S2 and S3 Figs). Animals in long-term experiments were immunized three times biweekly, and blood samples were collected before each immunization and monthly until 24 weeks. Antibody titers against the immunizing peptide were determined with enzyme-linked immunosorbent assay (ELISA).

### Plasma levels of mouse PCSK9 (muPCSK9) and target engagement

Plasma muPCSK9 concentration was determined with a Mouse Proprotein Convertase 9/PCSK9 Immunoassay, Quantikine^®^ ELISA (R&D SYSTEMS, Inc., Minneapolis, MN, USA, MPC900), according to the manufacturer’s instructions.

### Plasma levels of lipoprotein profiles and biochemical analysis

Plasma was acquired via centrifugation and stored at -80°C until measurement. A 35-μl plasma sample was used for lipoprotein profiling with high-performance liquid chromatography and molecular sieve columns with a LipoSEARCH system (Skylight Biotech, Inc., Tokyo, Japan) to measure the total cholesterol (TC), very low-density lipoprotein (VLDL), LDL, high-density lipoprotein (HDL), chylomicron (CM), and triglyceride (TG) content [7]. A 100-μl plasma was used for analysis to determine total protein (TP), Globulin (GLOB), alanine aminotransferase (ALT), alkaline phosphatase (ALP), total bilirubin (TBIL), blood urea nitrogen (BUN), creatinine (CRE) and electrolytes levels by using an Automatic Biochemical Analyzer (VetScan VS2, ABAXIS).

### Hepatocyte cell surface expression levels of LDLR

For the tissue analysis of vaccinated mice, they were sacrificed after subcutaneous injection of pentobarbital (200 mg/kg). Mouse liver tissue (100 mg) was homogenized in 500 μl RIPA lysis buffer with 1x PMSF, sodium orthovanadate and protease inhibitors (Santa Cruz Biotechnology, Inc., Dallas, TX, USA, sc-24948) on ice and centrifuged at 10,000 × g for 30 minutes as described elsewhere [8]. LDLR concentration were calculated using a Mouse LDLR Immunoassay in the Quantikine^®^ ELISA kit (R&D SYSTEMS, Inc., Minneapolis, MN, USA, MLDLR0), according to the manufacturer’s instructions.

### Histological analysis

The isolated kidney and lung were fixed in 4% paraformaldehyde for 24 hours and sliced in 4-μm sections. The sections were reacted with primary antibody (Abcam plc, Cambridge, UK, ab-6640; rat monoclonal anti-F4/80 antibody [1:8000 dilution]) and secondary antibody (Vector Laboratories, US, biotinylated anti-rat IgG). To detect the immune complex, the sections were directly reacted with secondary antibody (Nichirei Corporation, Tokyo, Japan, 414321; biotinylated anti-mouse polyclonal IgG) without treating primary antibody after blocking with 3% BSA. The sections were counterstained with hematoxylin and mounted for microscopic observation (Olympus FSX100, Japan).

### Enzyme-linked immunosorbent assay (ELISA)

PCSK9-specific antibody responses were measured by coating ELISA plates with bovine serum albumin-PCSK9 conjugate or recombinant mouse PCSK9 (Abcam plc, Cambridge, UK, ab-167759). Candidate PCSK9 peptides were conjugated with bovine serum albumin at its N-terminus via suberic acid bis (PEPTIDE INSTITUTE Inc., Osaka, Japan). The bovine serum albumin-PCSK9 conjugate or mouse recombinant PCSK9 was coated onto ELISA plates at 10 μg/mL in carbonate buffer overnight at 4°C. Serum samples from immunized mice were blocked with a 5% skim milk solution in phosphate-buffered saline (PBS), diluted from 100- to 325,000-fold in PBS containing 5% skim milk and incubated overnight at 4°C. Samples were washed with PBS containing 0.05% Tween 20 (PBS-T), and plates were incubated with horseradish peroxidase-conjugated antibodies specific for mouse IgG (GE Healthcare Life Sciences, Pittsburgh, PA, USA) for 3 h at room temperature. Anti-mouse IgG subclass-specific horseradish peroxidase-conjugated antibodies (IgG1, IgG2b, and IgG2c) were used for the IgG subclass determination assay. Plates were washed with PBS-T, and color was developed with the peroxidase chromogenic substrate 3,3′-5,5′-tetramethyl benzidine (Sigma-Aldrich Co., St Louis, MI, USA). The reaction was stopped using 0.5 N sulfuric acid. Absorbance was detected using a microplate reader (Bio-Rad Laboratories, Inc., Hercules, CA, USA) at 450 nm. The half-maximal antibody titer was determined according to the highest value in the dilution range of each sample.

### ELISPOT assay

These assays were performed as described previously [9]. Briefly, 96-well ELISPOT plates (EMD Millipore, Billerica, MA, USA) were coated with an anti-mouse interferon-gamma (IFN-γ) capture antibody or anti-mouse interleukin-4 (IL-4) capture antibody overnight at 4°C. The plates were washed with a PBS-T solution and blocked with 1% bovine serum albumin and 5% (wt/vol) sucrose in PBS. Splenocyte suspensions from V2 vaccine-immunized mice were added to the plates (10^6^ cells per well) and stimulated with 10 μg/mL candidate PCSK9 peptide, recombinant mouse PCSK9 protein (Abcam plc, Cambridge, UK, ab167759), KLH, and phytohemaggulutinin (PHA) at 37°C for 48 h. The plates were washed with PBS-T after incubation with a biotinylated anti-mouse IFN-γ or IL-4 antibody overnight at 4°C. Plates were washed, and streptavidin-alkaline phosphatase was added to each well and incubated for 2 h at room temperature. Plates were washed with PBS-T and incubated with a 5-bromo-4-chloro-3 indolylphosphate P-toluidine salt and nitro blue tetrazolium solution for 30 min at room temperature. Plates were rinsed with water and air-dried at room temperature. Colored spots were quantified using a dissecting microscope (Leica Microsystems, Wetzlar, Germany, LMD6500).

### Statistical analysis

All values are expressed as the means ± SEM. Differences between two groups were assessed using unpaired two-tailed Student’s t tests. Datasets involving more than two groups were analyzed by one-way ANOVA or a 2-factor repeated-measure ANOVA. Post hoc analyses were performed with Tukey’s multiple comparisons test. P values less than 0.05 were considered statistically significant. All statistical analyses were performed using Prism version 5.01 (GraphPad Software, Inc., La Jolla, CA, USA).

## Results

### Selection and screening of the antigen sequence for the anti-PCSK9 vaccines

**Fig 1:**
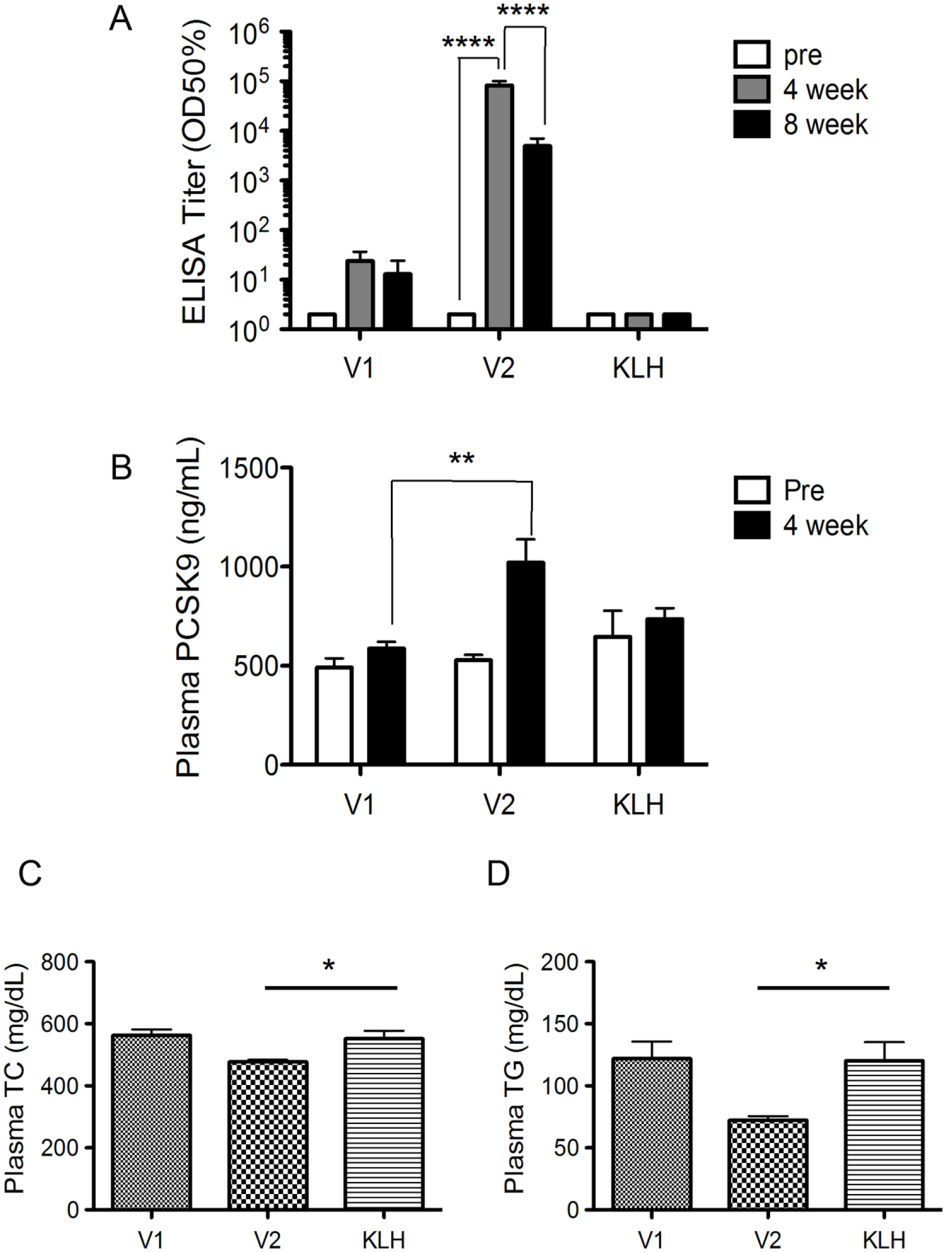
Screening of PCSK9 peptide vaccines in male *ApoE-deficient mice*. Two candidate vaccines (V1 and V2 vaccines) or control (KLH) was injected (5 μg peptide per mouse) (N = 4 per group). (A) The antibody titers against candidate PCSK9 peptides conjugated with bovine serum albumin were evaluated pre-immunization (pre) and post-immunization (4 or 8 weeks), and the results are expressed as half-maximal binding (optical density: OD50%). Significance values were obtained with a 2-factor repeated-measure ANOVA with subsequent Tukey’s multiple comparisons tests. (B) Mouse plasma PCSK9 levels were measured at pre-immunization (pre) and post-immunization (4 weeks) time points. Significance values were obtained using two-way ANOVA with subsequent Tukey’s multiple comparisons test. (C and D) Mean values of TC and TG levels (mg/dL) were measured post-immunization (4 weeks). Significance values relative to KLH (*P<0.05) were obtained with one-way ANOVA with subsequent Tukey’s multiple comparisons tests. All data in this Figure are expressed as the means ± SEM. *P<0.05, **P<0.01, and ****P<0.0001.

The peptide vaccines for mouse PCSK-9 consisted of a short peptide sequence conjugated to keyhole limpet hemocyanin (KLH) as a carrier protein. The three-dimensional structure and epitope information of PCSK-9 [10] allowed for the selection of two short antigens (9–10 amino acids in length) in the C-terminal cysteine/histidine-rich domain of the PCSK9 protein (V1, 580–589 aa; V2, 682–690 aa). Initially, the vaccines (5 μg peptide per mouse) were administered twice in 2-week intervals to male *ApoE-deficient mice* (11 weeks of age; n = 4) (S2 Fig). The vaccine generated by epitope V2 (V2 vaccine) elicited a significant antibody response against candidate PCSK9 peptides conjugated with bovine serum albumin 4 weeks after the first immunization. The V1 vaccine and the control vaccine (containing only with the carrier protein KLH) did not exhibit a marked increase in anti-PCSK9 antibody titer. Antibody titers gradually decreased 8 weeks after the first immunization, but these titers tended to remain higher in the V2 vaccine group (Fig 1A). We further investigated the ability of the induced antibodies from the V1 and V2 vaccine groups to bind the recombinant PCSK9 protein. V1 and V2 vaccine groups elicited a significant antibody response to recombinant mouse PCSK9 protein (P<0.05 and P<0.01, respectively), as compared with the response in the KLH control group (S4A Fig).

Previous studies have revealed that the presence of anti-PCSK9 antibodies increases total soluble PCSK9 levels in the plasma [8, 11], thus suggesting that anti-PCSK9 antibodies engage with PCSK9 in vivo and form an immune complex. Plasma PCSK9 levels were measured in both vaccine groups prior to vaccination and 4 weeks after the first immunization to evaluate this phenomenon. Plasma PCSK9 levels increased significantly in mice immunized with the V2 vaccine compared to in mice immunized with the V1 vaccine at 4 weeks after the first immunization (1020.6 ± 117.3 ng/mL vs. 586.3 ± 34.77 ng/mL; P <0.01) (Fig 1B). Plasma TC and triglyceride (TG) were measured in mice immunized with V1 and V2 vaccines and mice that received the KLH control to assess the effect of the PCSK9 vaccine. The results indicated that plasma TC and TG levels decreased significantly in the V2 vaccine group compared with the V1 vaccine group or the KLH control group (P<0.05) (Fig 1C and 1D).

Considering the clinical application of immunotherapy for human, we also performed the additional experiment on our V2 vaccine with alum as a human adjuvant. As a result, even in the vaccine to which alum was conjugated, remarkable increase in antibody titers were observed as in the case of Freund’s adjuvant (S4B Fig). On the basis of these results, V2 vaccine was selected and further evaluated in additional and long-term experiments.

### Assessment of dose-dependency and long-term efficacy of the V2 vaccine

A low (5 μg peptide per mouse) or high (50 μg peptide per mouse) dose of the V2 vaccine was administered in combination with Freund’s adjuvant to male *ApoE-deficient mice* (11 weeks of age; n = 7) to evaluate the dose-dependency. Vaccines were administered to mice three times with a 2-week interval between injections (S3 Fig). The antibody titer against PCSK9 6 or 8 weeks after the first immunization increased significantly in the low- and high-dose vaccine groups relative to pre-immunization, but not in the control group (mice administered only saline) (S5 Fig). However, there were no significant changes in antibody titers between low-dose and high-dose groups.

**Fig 2:**
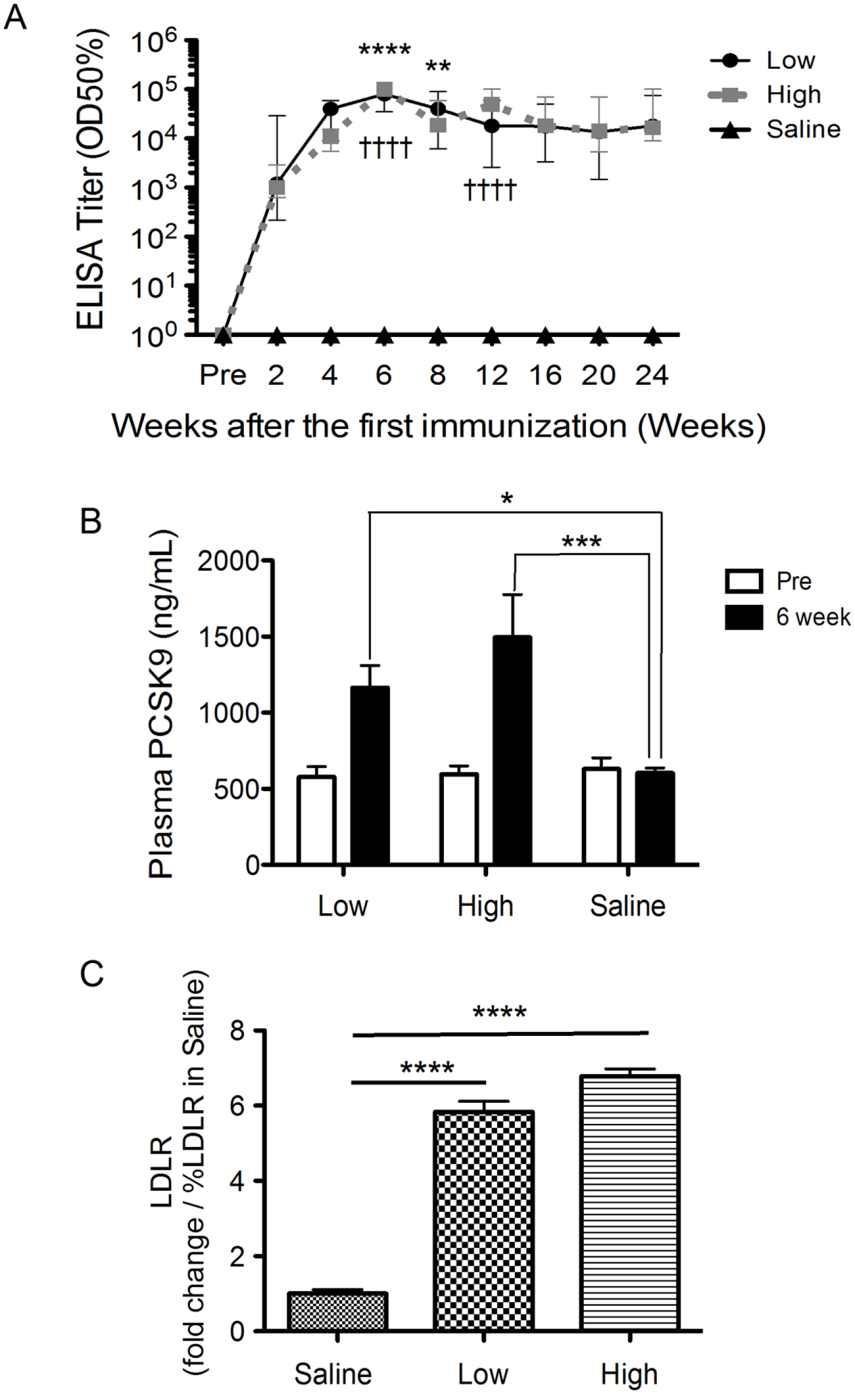
Evaluation of the PCSK9 vaccine (V2 vaccine) in male *ApoE-deficient mice* (N = 5–7). PCSK9 vaccine (V2 vaccine) or control (Saline) was injected at different doses (Low; 5 μg and High; 50 μg peptides per mouse) three times in biweekly intervals (0, 2, and 4 weeks). (A) Anti-PCSK9 antibody titers (OD50%) were evaluated at pre-immunization (pre) and post-immunization time points (2, 4, 6, 8, 12, 16, 20, and 24 weeks). Data are presented as the average of each groups; error bars indicate the SEM. **P<0.01 and ****P<0.0001 show significant changes between low dose group and saline group. ††††P<0.0001 shows significant changes between high dose group and saline group. (B) PCSK9 levels in plasma samples from pre-immunized (pre) and post-immunized (6 weeks) mice. Bars represent mean levels of detected mouse PCSK9 levels in plasma samples, and error bars represent ± SEM. Significance values relative to saline group (*P<0.05, ***P<0.001) were obtained using two-way ANOVA with subsequent Tukey's multiple comparisons tests. (C) Cell-surface LDLR levels in liver hepatocytes were measured in immunized mice at 6 weeks post-immunization via ELISA. The results are presented as a fold-increase relative to saline-treated groups. Significance values relative to saline (****P<0.0001) were obtained with one-way ANOVA with subsequent Tukey’s tests for multiple comparisons. All data in this Figure are expressed as the means ± SEM.

PCSK9-specific immunotherapy using monoclonal antibodies results in a clear decrease in TC [12]. However, the in vivo half-lives of these monoclonal antibody therapies limit the duration of the effect. We performed longer-term studies to assess the long-term efficacy of the V2 vaccine. *ApoE-deficient mice* were vaccinated three times with a 2 week interval between injections, and we monitored and evaluated antibody titers and lipoprotein levels up to 24 weeks after the first immunization (S3 Fig). Anti-PCSK9 antibody titers in both low- and high-dose vaccine groups reached peak levels (OD50% = 100,000) and increased significantly relative to saline group 6 weeks after the prime immunization, and these titers (OD50%>10,000) were maintained for up to 24 weeks (Fig 2A).

Plasma levels of PCSK9 were measured prior to the first vaccination (pre) and 6 weeks after the first vaccination. As described above, anti-PCSK9 antibody treatment increased plasma PCSK9 levels. In Fig 2B, we found that plasma levels of PCSK9 increased significantly in low-dose and high-dose groups relative to saline group at 6 week after the first vaccination (P<0.05 and P<0.001, respectively) (Fig 2B). PCSK9 synthesized in the liver interacts with the LDLR [13], thus leading to its internalization and subsequent lysosomal degradation [14, 15, 16] and the subsequent down-regulation of the number of cell-surface LDLR molecules [17]. The induced antibodies decrease PCSK9’s interaction with the LDLR and internalization, and an increase in LDLR expression was expected. Cell-surface LDLR levels in hepatocytes of immunized mice were analyzed to confirm this hypothesis. Mice immunized with low- or high-dose V2 vaccine exhibited a 6- and 7-fold increase, respectively, in cell-surface LDLR expression compared with the percentage LDLR in negative controls (P<0.0001) (Fig 2C).

**Fig 3:**
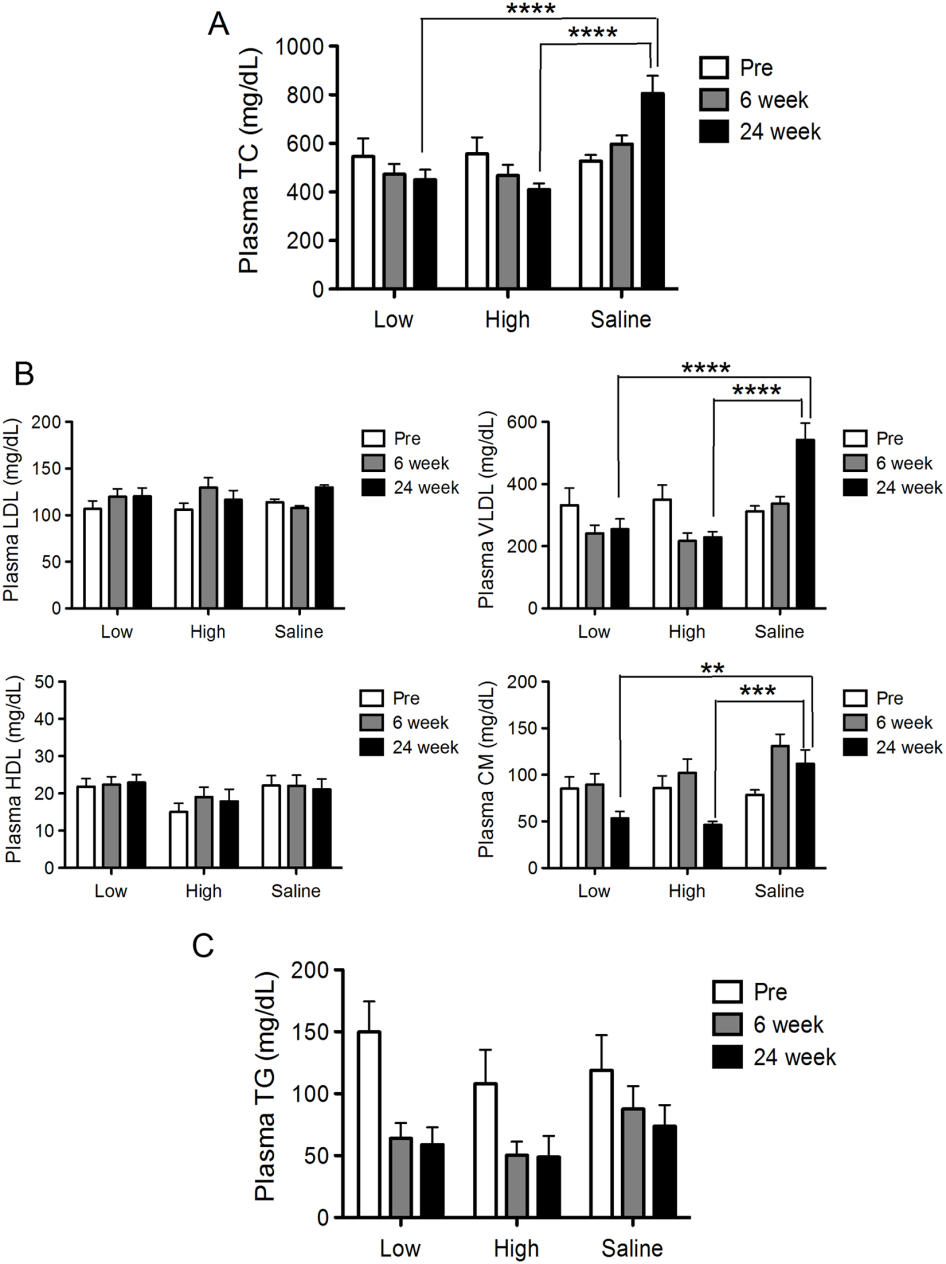
Evaluation of long-term efficacy of PCSK9 vaccine (N = 5–7). PCSK9 vaccine (V2 vaccine) was injected at a low dose (5 μg) or high dose (50 μg) peptide per mouse three times in biweekly intervals (0, 2, and 4 weeks), and mice were followed up until 24 weeks. Plasma TC levels (A), detailed cholesterol lipoprotein profile (B), and plasma TG levels (C) were measured at the particular time points (pre-immunization, 6 and 24 weeks after first immunization). Significance values were obtained with a 2-factor repeated-measure ANOVA with subsequent Tukey’s multiple comparisons tests. All data in this Figure are expressed as the means ± SEM. **P<0.01 ***P<0.001, and ****P<0.0001.

We further assessed the plasma levels of lipoprotein profiles in order to evaluate the efficacy of PCSK9 vaccine. At 24 weeks after the first vaccination, the levels of TC in both low- and high-dose group was significantly decreased than that in saline group (Fig 3A). In addition, we performed the detailed cholesterol lipoprotein profile analyses to measure LDL, very low-density lipoprotein (VLDL), and high-density lipoprotein (HDL) cholesterol and chylomicron (CM) levels. The high-dose group and, to a lesser extent, the low-dose group significantly decreased VLDL and CM in plasma at 24 weeks (Fig 3B). No significant differences in the levels of HDL, LDL and TG were detected between any dose and weeks groups (Fig 3C).

### Evaluation of T-cell activation in anti-PCSK9 vaccinated mice

**Fig 4:**
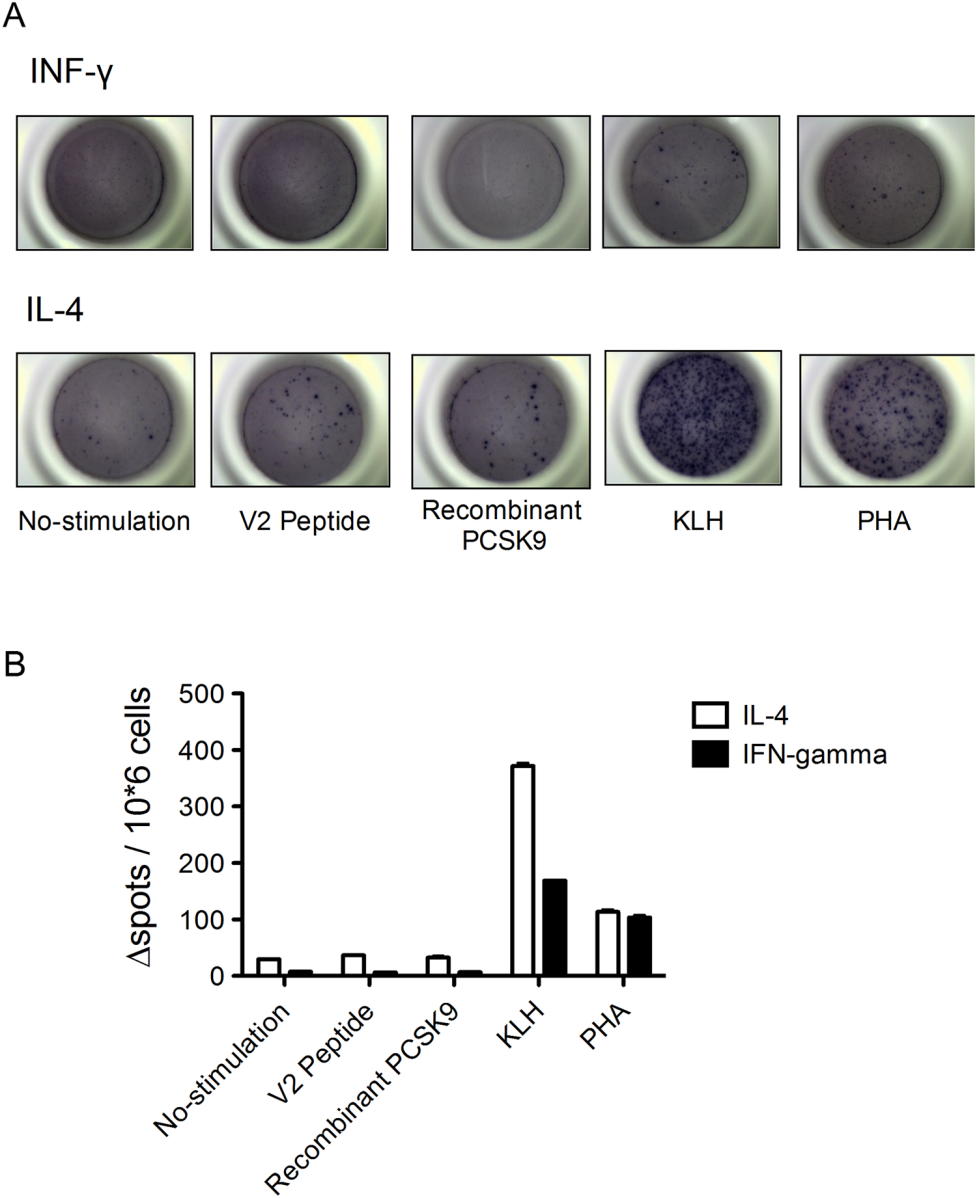
Evaluation of T-cell activation by using ELISpot assays in vaccinated mice (N = 5–7). Splenocytes (10^6^ cells per well) from V2 vaccine-immunized mice were stimulated with a candidate PCSK9 peptide (V2 peptide), recombinant mouse PCSK9, KLH, or PHA at 10 μg/mL. (A) Representative images of black spots are shown detecting INF-γ and IL-4 in splenocytes. (B) Cytokine (IFN-γ and IL-4)–producing cells were quantified. All data are expressed as the means ± SEM. Significance values were obtained using two-way ANOVA with subsequent Tukey’s multiple comparisons tests.

Our therapeutic vaccination was generated against self-proteins. Therefore, it was necessary to consider safety, such as the absence of a target-specific T-cell response (T-helper [Th] and cytotoxic T-cells) [18]. Using an ELISpot assay, we evaluated the production of IFN-γ and interleukin-4 (IL-4) cytokines, which are associated with Th1-type and Th2-type responses, respectively, in splenocytes, from mice immunized with the V2 vaccine. Stimulation with KLH induced IFN-γ and IL-4 production and generated significant numbers of splenocytes that produced both cytokines in similar levels as those produced by splenocytes treated with the T-cell mitogen phytohemagglutinin (PHA) as a positive control. However, V2 peptide, which contained a candidate epitope in the V2 vaccine, and recombinant mouse PCSK9 protein did not elicit a significant response, a result identical to that in the no stimulation group (Fig 4A and 4B). These results indicated that KLH contained adequate T-cell epitopes to induce T-cell activation in immunized mice, and PCSK9 did not elicit T-cell activation even after immunization with V2 peptide. Therefore, the selected peptide used for immunization did not constitute a T-cell epitope and lead to the induction of PCSK9-specific T-cells. Most T cells differentiated into Th2-type cells after V2 vaccination, thus suggesting that this level of T-cell activation was sufficient to promote antibody production but not induce an autoimmune response. We also evaluated their IgG subclass of induced anti-PCSK9 antibodies. Because the production of IgG1 antibodies is primarily induced via Th2-type cytokines, and the production of IgG2b antibodies reflects the involvement of Th1-type cytokines. [19]. The IgG subclass enzyme-linked immunosorbent assay (ELISA) demonstrated that the ratio of IgG1 to IgG2b antibodies in mice immunized with V2 vaccine was greater than 1.0, thus indicating that the V2 vaccine induced a primarily Th2-type response (S6A Fig).

In addition, to establish the safety of our anti-PCSK9 peptide vaccine, we performed the plasma biochemical analysis and pathological analysis of the kidney and lung. As a result of blood examination in clinical practice (electrolytes, and liver and kidney function), there were no significant differences among those experimental groups (S7 Fig). Furthermore, in pathological analysis, we could not find any infiltration of macrophage cells (stained with anti-F4/80 antibody) and immune complexes (stained with anti-mouse IgG) in lungs and kidneys of mice immunized with our vaccine (S8 Fig).

## Discussion

In this study, we developed an anti-PCSK9 peptide vaccine to increase the levels of plasma PCSK9 and the expression of cell-surface LDLR, and to improve plasma lipoprotein profiles. We further demonstrated the efficacy of the vaccine for dyslipidemia in improving the dyslipidemic phenotype without causing adverse autoimmune responses.

Anti-PCSK9 therapeutic vaccines have been found to decrease LDL cholesterol levels via inhibition of PCSK9-LDLR interactions [11, 20]. However, the selected epitope from previous studies is quite different from that of our vaccine. PCSK9 protein is composed of an N-terminal prodomain, a subtilisin-like catalytic domain and a C-terminal cysteine/histidine-rich domain. PCSK9 was crystallized in a complex with the epidermal growth factor-like repeat A domain of the LDLR at acidic and neutral pH values [21]. Notably, the LDLR epidermal growth factor-like repeat A domain binds exclusively to the catalytic domain of PCSK9. Therefore, previous vaccines have selected epitopes from this catalytic domain [11, 20]. Notably, the PCSK9 C-terminal domain is not directly involved in PCSK9-LDLR binding [22], but human genetic analyses of FH have suggested that several gain- (H553R) or loss- (S462P and Q554E) point mutations within the C-terminal domain of PCSK9 result in hyper- or hypocholesterolemia, respectively [23–26]. These data support the functional significance of the C-terminal domain in the PCSK9-LDLR interaction. Ni YG et al. have also demonstrated that the PCSK9 C-terminal domain contributes to inhibition of LDLR function primarily via effects on the cellular uptake of PCSK9 and LDLR complex without altering the binding affinity for LDLR [27]. These studies have also revealed that the lack of the C-terminal domain compromises the ability of PCSK9 to internalize into cells and inhibit LDL uptake. Our selected vaccines also demonstrated that a sustained TC, VLDL, and CM decrease and up-regulated LDLR expression was achieved for up to 6 months, a result consistent with the previous findings. This study is the first vaccination approach using PCSK9 inhibition targeting of the C-terminal domain.

For the evaluation of lipid metabolism or atherosclerosis, male *ApoE-deficient mice* was usually used [28], because estogens strongly influence lipid and cholesterol metabolism in female mice. Thus, female mice need to be subjected to overiectomy in order to evaluate the effect of our anti-PCSK9 vaccine on lipid metabolism. In addition, there are some reports that atherosclerotic lesions in *ApoE-deficient mice* increased with ages and more in male mice [29]. Furthermore, Roubtsova A et al reported that the absence of PCSK9 results in a sex- and tissue-specific subcellular distribution of the LDLR and VLDLR, which is determined by 17β-estradiol levels [30]. In an effort to limit the influence associated with technical and complex strategy, we selected only male *ApoE-deficient mice* in this study. In line with these observations, our approach increased the expression of cell-surface LDLR in hepatocytes which consequently resulted in an improvement of TC, especially VLDL, in male *Apo-E deficient mice*. The phenotype of *ApoE-deficient mice* includes a hyperlipidemic plasma profile with disrupted hepatic uptake of both liver- and intestine- derived ApoB-containing lipoproteins, thus resembling human hyperlipoproteinemia type III [31, 32]. Plasma cholesterol in this model is carried in ApoB-48-containing VLDL particles and chylomicron remnants, and *ApoE-deficient mice* contain high levels of VLDL in their lipoprotein profiles. Up-regulation of LDLR in hepatocytes generally promotes plasma cholesterol uptake via LDLR ligands, primarily ApoB and ApoE, thus leading to a decrease in these factors in the blood circulation. However, the levels of LDL cholesterol are extremely high in *ApoE-deficient mice*, because ApoB-48 lacks the LDLR binding site in the C terminus of the protein [33]. This study did not observe a decrease in LDL cholesterol levels despite the up-regulation of LDLR expression. Instead, a significant decrease in VLDL levels was observed. We hypothesized that ApoB is essential for the assembly and secretion of nascent VLDL particles [34] and that the post-translational degradation of ApoB-48 promoted by LDLR expression decreases VLDL particle secretion. Indeed, studies in humans, mice, and primary hepatocytes have revealed that a loss of LDLR activity increases the secretion of VLDL particles because of a decrease in ApoB degradation [35–37]. The anti-PCSK9 antibodies produced by our selected vaccine target inhibited PCSK9-LDLR internalization and up-regulated cell-surface LDLR expression, thereby influencing the plasma levels of VLDL particles. In contrast, we did not find significant changes in HDL particles in *ApoE-deficient mice*. Most HDL particles in humans do not contain ApoE or bind to the LDLR. Therefore, the effect of blocking PCSK9-LDLR internalization using vaccination would result primarily in a decrease in VLDL cholesterol. Although the lipid profile changes resulting from anti-PCSK9 therapy may be different in humans, the PCSK9 vaccine had beneficial effects on lipid profile control in mice.

When considering the clinical application of immunotherapy for dyslipidemia, the goal of therapeutic vaccines targeting self-antigen is to keep the antibody titers that effectively and specifically neutralized targeting protein without concomitantly inducing a target-specific T cell activation. The antigens generally contain B- and T-cell epitopes to induce the antibody production, because the helper T cell activation is required to assist the expansion of B-cells. The effectiveness and safety of vaccines depend on the appropriate activation between T cell and B cell by specific epitopes within the peptide sequences of our target protein. Some studies illustrated that only a low level of sequence similarity to the host proteome is needed to module the B cell epitope-specific peptidome [38, 39]. In this study, we selected two short (9–10 amino acids) peptide (V1 and V2 peptide) as candidate targets for our anti-PCSK9 vaccine and conjugated these peptides to KLH which presents a variety of T cell epitopes to induce helper T-cell responses. Indeed, T cell activation analysis using ELISPOT assay provided information that splenocytes from PCSK9 vaccinated mice stimulated with KLH produced IFN-γ and IL-4, however splenocytes stimulated with the V2 peptide did not produce these cytokines (Fig 4A and 4B). Furthermore, we examined what happens when mice are immunized with recombinant PCSK9 instead of peptide alone. As a result, we could not find the anti-PCSK9 antibody titers (S6B Fig). These results indicated that KLH contains adequate T-cell epitopes to induce T-cell activation, whereas V2 peptide did not elicit T-cell activation after immunization with V2 vaccine, comfirming V2 vaccine as an exclusive B cell antigen. Furthermore, we confirmed the safety of V2 vaccine by biochemical and histological analysis of the kidney and lung after immunization (S7 and S8 Figs).

Theoretically, there are some limitation in our study. In this study, it was a half-year observation period and the boost shot to maintain the anti-PCSK9 antibody production was not done because high antibody titers and effectiveness to decrease lipoprotein lasted during that period. However, recurring immunization and further evaluation of the safety and the opposite-tolerance to the antigen are required to support the clinical application of future vaccines.

In summary, we produced an anti-PCSK9 vaccine targeting the C-terminal domains as an antigen epitope. Our anti-PCSK9 vaccine induced long-lasting anti-PCSK9 antibody production, up-regulated cell surface LDLR expression and significantly decreased VLDL cholesterol and CM in *ApoE-deficient mice*. Therefore, anti-PCSK9 vaccination may be an innovative approach and may provide a new therapeutic option for the treatment of dyslipidemia.

## Supporting information

S1 Fig *ApoE-deficient mice* generated by using CRISPR-Cas9 gene editing. *ApoE-deficient mice* were created via the insertion of mutations in Exon4 of the apoE gene. The asterisk indicates the inserted thymine.
(PDF)

S2 Fig Time course of PCSK9 peptide vaccine in male *ApoE-deficient mice*. Two candidate vaccines (V1 and V2 vaccine) or control (KLH) was injected (5 μg peptide per mouse). (N = 4 for each group).
(PDF)

S3 Fig Time course of PCSK9 vaccine (V2) in male *ApoE-deficient mice*. It is shown for evaluating antibody titer, plasma PCSK9 and lipid profile. PCSK9 vaccine (V2 vaccine) or control (Saline) was injected at different doses (Low; 5 μg and High; 50 μg peptide per mouse).
(PDF)

S4 Fig Screening of PCSK9 peptide vaccine in male *ApoE-deficient mice* (N = 4). (A) Antibody titers against recombinant mouse PCSK9 protein 4 weeks after the first immunization were assayed with ELISA. Significance values relative to KLH (*P<0.05, **P<0.01) were obtained with one-way ANOVA with subsequent Tukey’s multiple comparison tests. *(B) 5μg of the V2 peptides* mixed with Freund’s (FA) or Alum adjuvant was administered to *mice*. Anti-PCSK9 antibody titers were measured at post-immunization (4 weeks) time points and are expressed as the dilution of serum to give half-maximal binding (optical density: OD50%) ± SE of the mean.
(PDF)

S5 Fig Evaluation of dose-dependency with PCSK9 vaccine (V2) in male *ApoE-deficient mice*. Each dose (5 μg, and 50 μg peptide per mouse) of the V2 vaccine was administered to *male ApoE-deficient mice*. Anti-PCSK9 antibody titers were measured at pre-immunization (pre) and post-immunization (6 and 8 weeks) time points and are expressed as the dilution of serum to give half-maximal binding (optical density: OD50%) ± SE of the mean. Significance values relative to pre-immunization (**P<0.01, ****P<0.0001) were obtained using a 2-factor repeated-measure ANOVA with subsequent Tukey’s multiple comparison tests.
(PDF)

S6 Fig Evaluation of the T cell activation with candidate peptides. (*A*) The total IgG, IgG1 (Th2), IgG2b (Th1), and IgG2c (Th1) profiles of the humoral immune response were measured in immunized mice. The diluted sera (1:1250) were quantified as absorbance at 450 nm. All data are expressed as the means ± SEM. Significance values were obtained with two-way ANOVA with subsequent Tukey’s multiple comparison tests. In this IgG subclass ELISA, the IgG1:IgG2b ratio in the anti-PCSK9 antibody pool was greater than 1.0 in the Vaccine 2 group (dilution 1:1250), thus indicating that vaccine 2 induced a primarily T helper (Th)2-type response (IgG1). (B) Only recombinant PCSK9 instead of our selected peptides (V2 peptide) were administered to *mice*. Anti-PCSK9 antibody titers were measured at post-immunization (4 weeks) time points and are expressed as the dilution of serum to give half-maximal binding (optical density: OD50%) ± SEM.
(PDF)

S7 Fig Biochemical analysis in male *ApoE-deficient mice with PCSK9 vaccine (V2 vaccine)* at 24 weeks. Blood samples was collected from the tail vein at 24 weeks after first-vaccination. Then, each plasma was acquired and used for biochemical analysis. There was no significant changes among each groups in all markers. Significance values were analyzed using one-way ANOVA with subsequent Tukey’s multiple comparison tests.
(PDF)

S8 Fig Pathological analysis in *apoE-deficient mice with PCSK9 vaccine (V2 vaccine)*. The section of kidney (left panel) and lung (right panel) of *apoE deficient* mice was prepared at 5 weeks after PCSK9 vaccine (V2 vaccine) injection. The representative pictures were shown, which was stained with anti-F4/80 antibody (upper panel) to evaluate the infiltration of macrophage cells and with anti-mouse IgG (lower panel) to detect the immune complex. Yellow bar indicates 100 μM.
(PDF)

S1 Table Statistics in Fig 1. (PDF)

S2 Table Statistics in Fig 2. (PDF)

S3 Table Statistics in Fig 3A and 3B. (PDF)

S4 Table Statistics in Fig 3B and 3C. (PDF)

S5 Table Statistics in Fig 4. (PDF)

S6 Table Statistics in S4 and S5 Figs. (PDF)

S7 Table Statistics in S6 Fig. (PDF)

S8 Table Test to analyze the Gaussian distribution for Figs 2B, 3A and 3B. [Kolmogorov-Smirnov (KS) test]. (PDF)
