# Supplementary material for: Development of vaccine for dyslipidemia targeted to a proprotein convertase subtilisin/kexin type 9 (PCSK9) epitope in mice
Source: PLoS One. 2018 Feb 13;13(2):e0191895. doi: 10.1371/journal.pone.0191895 (PMC5811007; doi:10.1371/journal.pone.0191895)
Supplement: S1 Fig — ApoE-deficient mice were created via the insertion of mutations in Exon4 of the apoE gene. The asterisk indicates the inserted thymine. (PDF) [file pone.0191895.s001.pdf]

S1 Fig

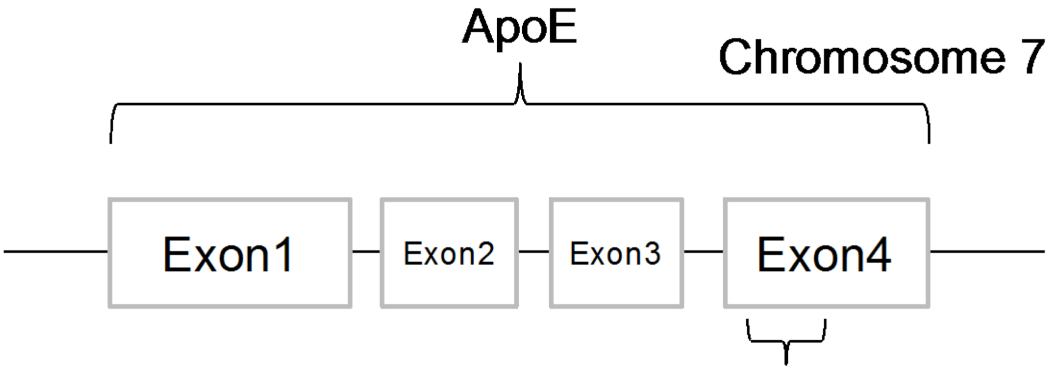

Cont GGCCTGCAGGGCACTGATGGAGGACACTATGACGGAAGTAAAGG

Mutant GGCCTGCAGGGCACTGATGGAGGACACTATGACGGAAGTTAAAG

[]

CTTACAAAAAGGAGC (Wild)

GCTTACAAAAAGGAGC (+1 bp)

S1 Fig. *ApoE-deficient mice* generated by using CRISPR-Cas9 gene editing. *ApoE-deficient mice* were created via the insertion of mutations in Exon4 of the apoE gene. The asterisk indicates the inserted thymine.
