# Supplementary material for: Development of vaccine for dyslipidemia targeted to a proprotein convertase subtilisin/kexin type 9 (PCSK9) epitope in mice
Source: PLoS One. 2018 Feb 13;13(2):e0191895. doi: 10.1371/journal.pone.0191895 (PMC5811007; doi:10.1371/journal.pone.0191895)
Supplement: S2 Fig — Two candidate vaccines (V1 and V2 vaccine) or control (KLH) was injected (5 μg peptide per mouse). (N = 4 for each group). (PDF) [file pone.0191895.s002.pdf]

S2 Fig

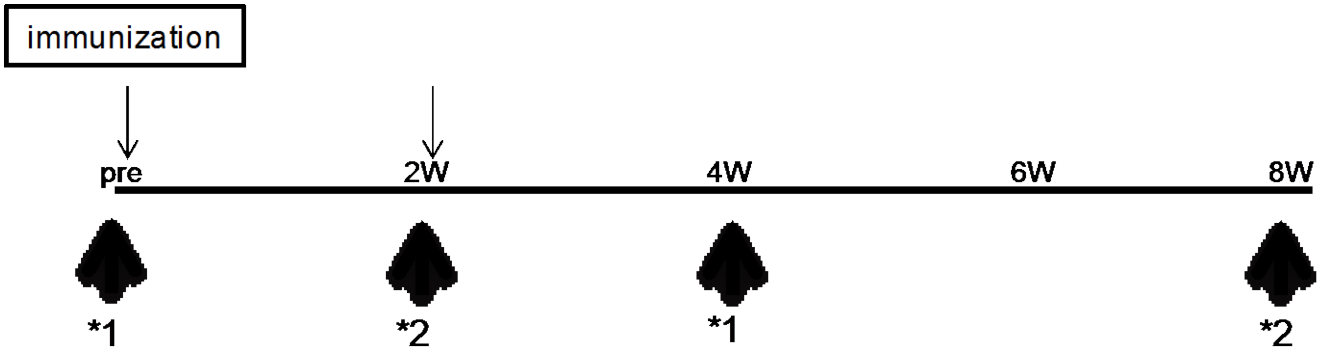

	Antibody Titer	Plasma PCSK9	Plasma Lipoprotein
*1	[]	[]	[]
*2	[]		

S2 Fig. Time course of PCSK9 peptide vaccine in male *ApoE-deficient mice*.

Two candidate vaccines (V1 and V2) or control (KLH) was injected (5 μg peptide per mouse). (N=4 for each group).
