# Supplementary material for: Development of vaccine for dyslipidemia targeted to a proprotein convertase subtilisin/kexin type 9 (PCSK9) epitope in mice
Source: PLoS One. 2018 Feb 13;13(2):e0191895. doi: 10.1371/journal.pone.0191895 (PMC5811007; doi:10.1371/journal.pone.0191895)
Supplement: S3 Fig — It is shown for evaluating antibody titer, plasma PCSK9 and lipid profile. PCSK9 vaccine (V2 vaccine) or control (Saline) was injected at different doses (Low; 5 μg and High; 50 μg peptide per mouse). (PDF) [file pone.0191895.s003.pdf]

S3 Fig

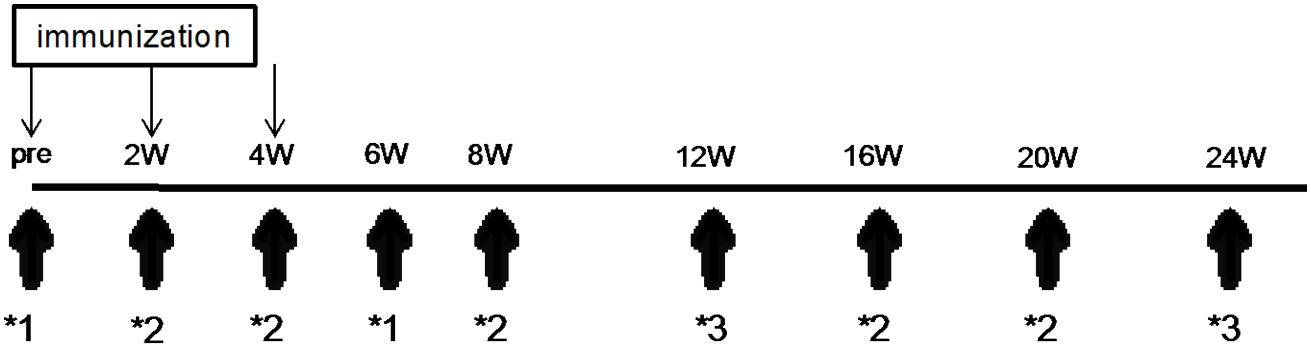

	Antibody Titer	Plasma PCSK9	Plasma Lipoprotein
*1	▯	▯	▯
*2	▯		
*3	▯		▯

S3 Fig. Time course of PCSK9 vaccine (V2) in male *ApoE-deficient mice.* It is shown for evaluating antibody titer, plasma PCSK9 and lipid profile. PCSK9 vaccine (V2) or control (Saline) was injected at different doses (Low; 5 μg and High; 50 μg peptide per mouse).
