# Supplementary material for: Development of vaccine for dyslipidemia targeted to a proprotein convertase subtilisin/kexin type 9 (PCSK9) epitope in mice
Source: PLoS One. 2018 Feb 13;13(2):e0191895. doi: 10.1371/journal.pone.0191895 (PMC5811007; doi:10.1371/journal.pone.0191895)
Supplement: S4 Fig — (A) Antibody titers against recombinant mouse PCSK9 protein 4 weeks after the first immunization were assayed with ELISA. Significance values relative to KLH (*P<0.05, **P<0.01) were obtained with one-way ANOVA with subsequent Tukey’s multiple comparison tests. (B) 5μg of the V2 peptides mixed with Freund’s (FA) or Alum adjuvant was administered to mice. Anti-PCSK9 antibody titers were measured at post-immunization (4 weeks) time points and are expressed as the dilution of serum to give half-maximal binding (optical density: OD50%) ± SE of the mean. (PDF) [file pone.0191895.s004.pdf]

S4 Fig

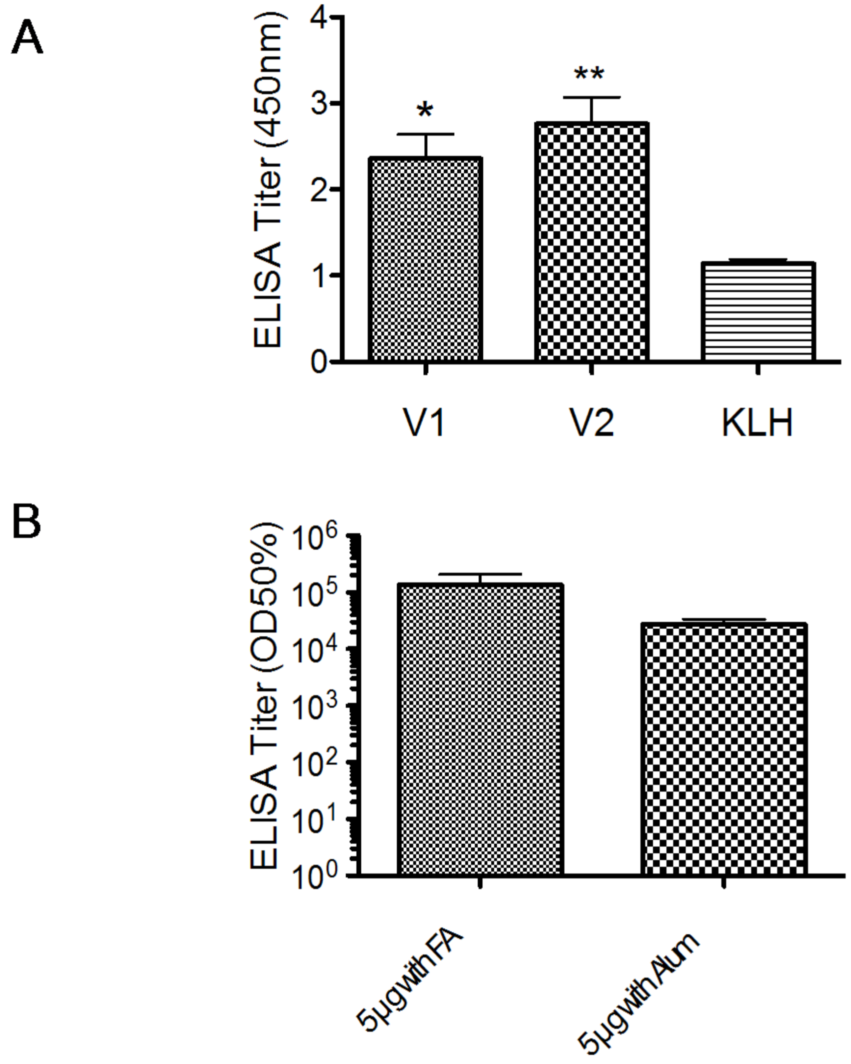

S4 Fig. Screening of PCSK9 peptide vaccine in male *ApoE-deficient mice* (N=4). (A) Antibody titers against recombinant mouse PCSK9 protein 4 weeks after the first immunization were assayed with ELISA. Significance values relative to KLH (*P<0.05, **P<0.01) were obtained with one-way ANOVA with subsequent Tukey's multiple comparison tests. *(B) 5μg of the V2 peptides* mixed with Freund's (FA) or Alum adjuvant was administered to *mice.* Anti-PCSK9 antibody titers were measured at post-immunization (4 weeks) time points and are expressed as the dilution of serum to give half-maximal binding (optical density: OD50%) ± SE of the mean.
