# Supplementary material for: Development of vaccine for dyslipidemia targeted to a proprotein convertase subtilisin/kexin type 9 (PCSK9) epitope in mice
Source: PLoS One. 2018 Feb 13;13(2):e0191895. doi: 10.1371/journal.pone.0191895 (PMC5811007; doi:10.1371/journal.pone.0191895)
Supplement: S5 Fig — Each dose (5 μg, and 50 μg peptide per mouse) of the V2 vaccine was administered to male ApoE-deficient mice. Anti-PCSK9 antibody titers were measured at pre-immunization (pre) and post-immunization (6 and 8 weeks) time points and are expressed as the dilution of serum to give half-maximal binding (optical density: OD50%) ± SE of the mean. Significance values relative to pre-immunization (**P<0.01, ****P<0.0001) were obtained using a 2-factor repeated-measure ANOVA with subsequent Tukey’s multiple comparison tests. (PDF) [file pone.0191895.s005.pdf]

S5 Fig

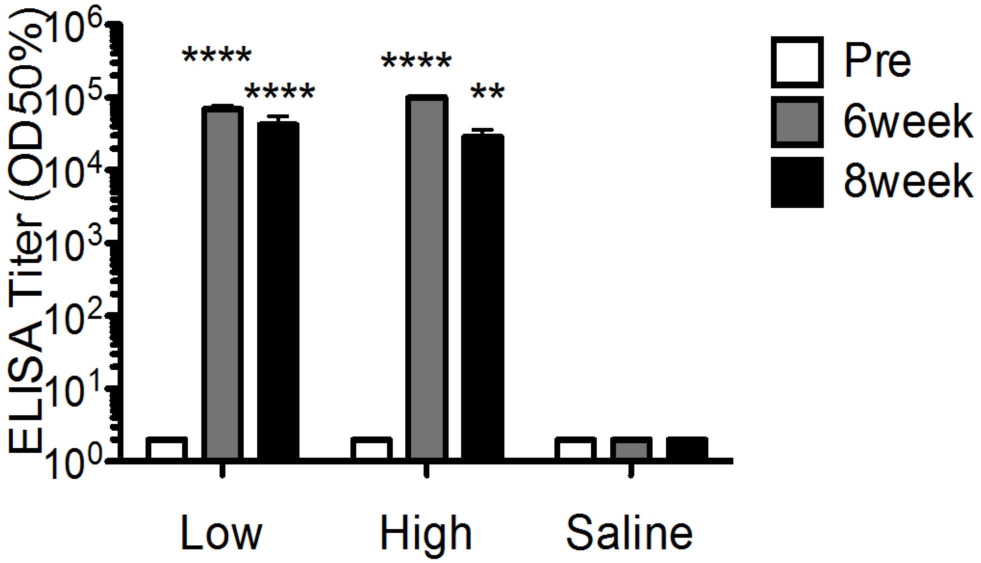

S5 Fig. Evaluation of dose-dependency with PCSK9 vaccine (V2) in male *ApoE-deficient mice*. Each dose (5 µg, and 50 µg peptide per mouse) of the V2 vaccine was administered to *male ApoE-deficient mice.* Anti-PCSK9 antibody titers were measured at pre-immunization (pre) and post-immunization (6 and 8 weeks) time points and are expressed as the dilution of serum to give half-maximal binding (optical density: OD50%) ± SE of the mean. Significance values relative to pre-immunization (**P<0.01, ****P<0.0001) were obtained using a 2-factor repeated-measure ANOVA with subsequent Tukey's multiple comparison tests.
