# Supplementary material for: Development of vaccine for dyslipidemia targeted to a proprotein convertase subtilisin/kexin type 9 (PCSK9) epitope in mice
Source: PLoS One. 2018 Feb 13;13(2):e0191895. doi: 10.1371/journal.pone.0191895 (PMC5811007; doi:10.1371/journal.pone.0191895)
Supplement: S6 Fig — (A) The total IgG, IgG1 (Th2), IgG2b (Th1), and IgG2c (Th1) profiles of the humoral immune response were measured in immunized mice. The diluted sera (1:1250) were quantified as absorbance at 450 nm. All data are expressed as the means ± SEM. Significance values were obtained with two-way ANOVA with subsequent Tukey’s multiple comparison tests. In this IgG subclass ELISA, the IgG1:IgG2b ratio in the anti-PCSK9 antibody pool was greater than 1.0 in the Vaccine 2 group (dilution 1:1250), thus indicating that vaccine 2 induced a primarily T helper (Th)2-type response (IgG1). (B) Only recombinant PCSK9 instead of our selected peptides (V2 peptide) were administered to mice. Anti-PCSK9 antibody titers were measured at post-immunization (4 weeks) time points and are expressed as the dilution of serum to give half-maximal binding (optical density: OD50%) ± SEM. (PDF) [file pone.0191895.s006.pdf]

S6 Fig

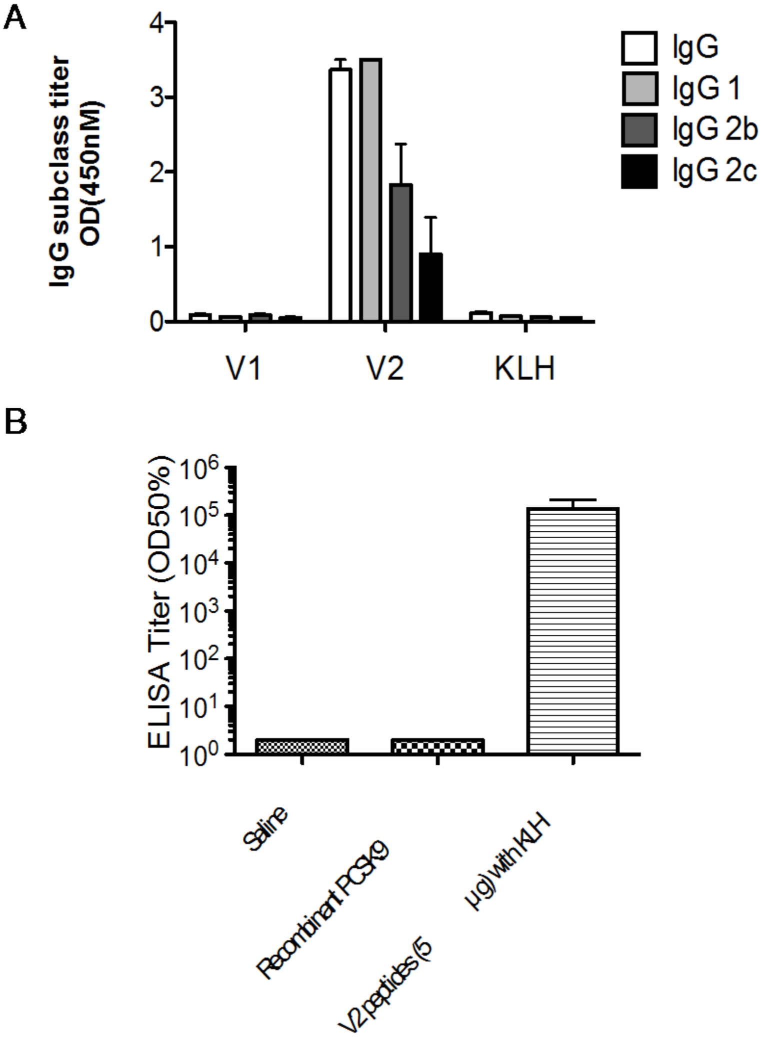

S6 Fig. Evaluation of the T cell activation with candidate peptides. (*A*) The total IgG, IgG1 (Th2), IgG2b (Th1), and IgG2c (Th1) profiles of the humoral immune response were measured in immunized mice. The diluted sera (1:1250) were quantified as absorbance at 450 nm. All data are expressed as the means ± SEM. Significance values were obtained with two-way ANOVA with subsequent Tukey's multiple comparison tests. In this IgG subclass ELISA, the IgG1:IgG2b ratio in the anti-PCSK9 antibody pool was greater than 1.0 in the Vaccine 2 group (dilution 1:1250), thus indicating that vaccine 2 induced a primarily T helper (Th)2-type response (IgG1). (B) Only recombinant PCSK9 instead of our selected peptides (V2 peptide) were administered to *mice.* Anti-PCSK9 antibody titers were measured at post-immunization (4 weeks) time points and are expressed as the dilution of serum to give half-maximal binding (optical density: OD50%) ± SEM.
