# Supplementary material for: Development of vaccine for dyslipidemia targeted to a proprotein convertase subtilisin/kexin type 9 (PCSK9) epitope in mice
Source: PLoS One. 2018 Feb 13;13(2):e0191895. doi: 10.1371/journal.pone.0191895 (PMC5811007; doi:10.1371/journal.pone.0191895)
Supplement: S7 Fig — Blood samples was collected from the tail vein at 24 weeks after first-vaccination. Then, each plasma was acquired and used for biochemical analysis. There was no significant changes among each groups in all markers. Significance values were analyzed using one-way ANOVA with subsequent Tukey’s multiple comparison tests. (PDF) [file pone.0191895.s007.pdf]

S7 Fig

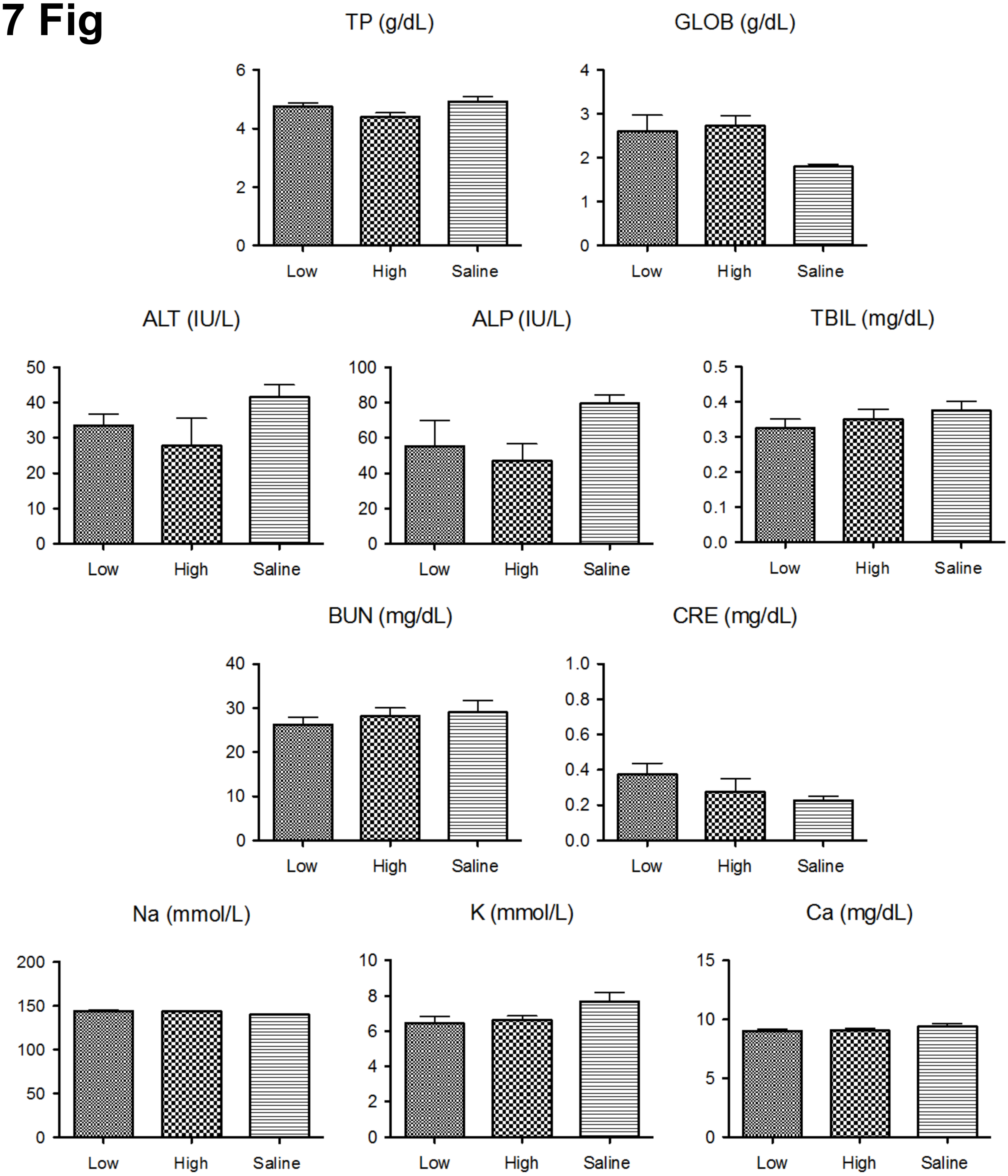

S7 Fig. Biochemical analysis in male *ApoE-deficient mice with PCSK9 vaccine (V2)* at 24 weeks. Blood samples was collected from the tail vein at 24 weeks after first-vaccination. Then, each plasma was acquired and used for biochemical analysis. There was no significant changes among each groups in all markers. Significance values were analyzed using one-way ANOVA with subsequent Tukey's multiple comparison tests.
