# Supplementary material for: Development of vaccine for dyslipidemia targeted to a proprotein convertase subtilisin/kexin type 9 (PCSK9) epitope in mice
Source: PLoS One. 2018 Feb 13;13(2):e0191895. doi: 10.1371/journal.pone.0191895 (PMC5811007; doi:10.1371/journal.pone.0191895)
Supplement: S8 Fig — The section of kidney (left panel) and lung (right panel) of apoE deficient mice was prepared at 5 weeks after PCSK9 vaccine (V2 vaccine) injection. The representative pictures were shown, which was stained with anti-F4/80 antibody (upper panel) to evaluate the infiltration of macrophage cells and with anti-mouse IgG (lower panel) to detect the immune complex. Yellow bar indicates 100 μM. (PDF) [file pone.0191895.s008.pdf]

S8 Fig

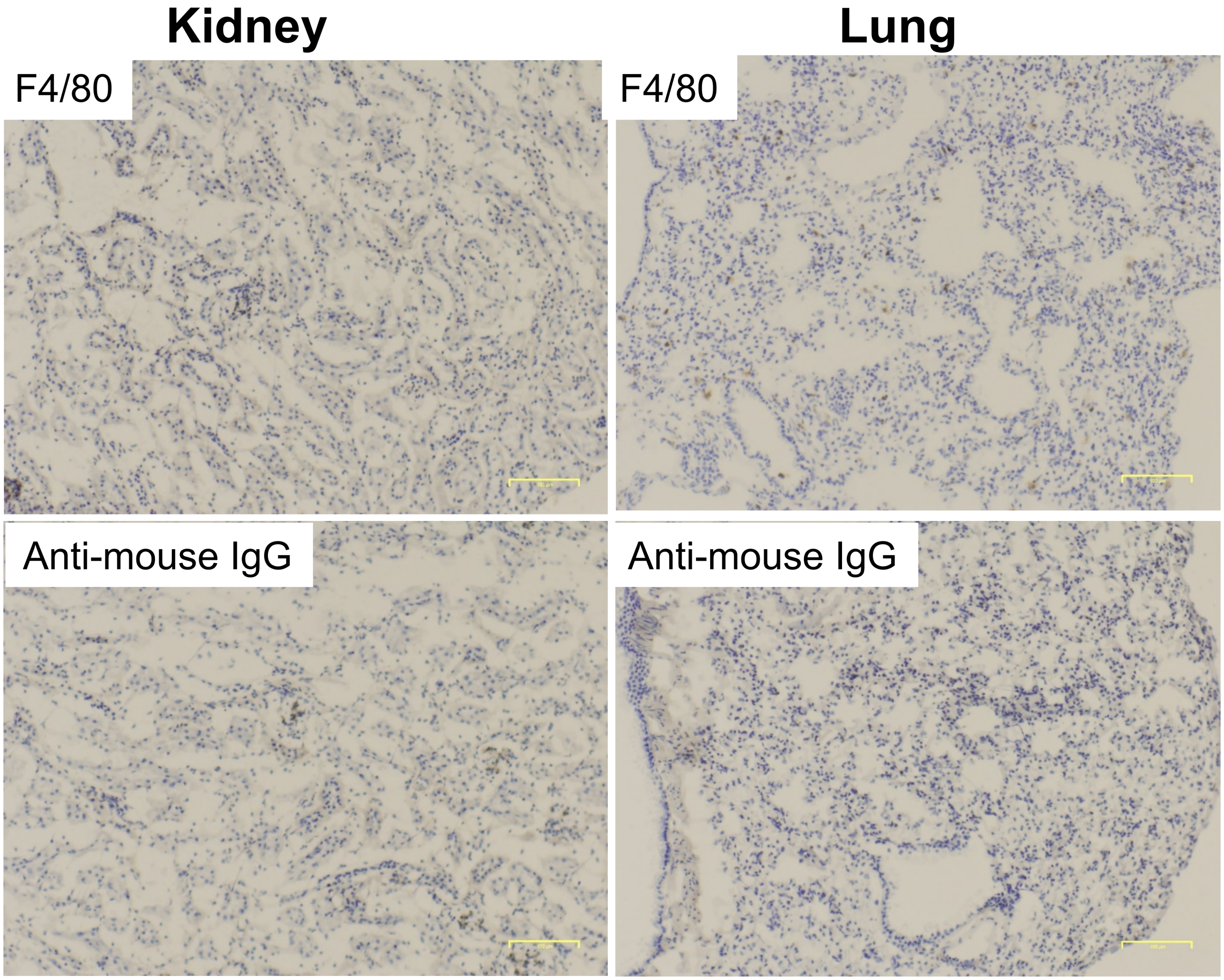

S8 Fig. Pathological analysis in *apoE-deficient mice with PCSK9 vaccine (V2 vaccine)*.

The section of kidney (left panel) and lung (right panel) of *apoE deficient* mice was prepared at 5 weeks after PCSK9 vaccine (V2 vaccine) injection. The representative pictures were shown, which was stained with anti-F4/80 antibody (upper panel) to evaluate the infiltration of macrophage cells and with anti-mouse IgG (lower panel) to detect the immune complex. Yellow bar indicates 100 μM.
