# Supplementary material for: Development of vaccine for dyslipidemia targeted to a proprotein convertase subtilisin/kexin type 9 (PCSK9) epitope in mice
Source: PLoS One. 2018 Feb 13;13(2):e0191895. doi: 10.1371/journal.pone.0191895 (PMC5811007; doi:10.1371/journal.pone.0191895)
Supplement: S1 Table — (PDF) [file pone.0191895.s009.pdf]

S1 Table. Statistics in Figure 1

	Two-way ANOVA	F (DFn, DFd)	P value
Fig. 1A	Interaction	F (4, 18)=20.67	P <0.0001
	Week	F (2, 18)=24.57	P <0.0001
	Treatment	F (2, 9)=20.71	P =0.0004
	Subject (matching)	F (9, 18)=0.9987	P =0.4750
**Tukey's multiple comparisons test**			
V1	Pre vs 4 week	Pre vs 8 week	4 week vs 8 week
**P value**	ns	ns	ns
V2	Pre vs 4 week	Pre vs 8 week	4 week vs 8 week
**P value**	<0.0001	ns	<0.0001
KLH	Pre vs 4 week	Pre vs 8 week	4 week vs 8 week
**P value**	ns	ns	ns

	Two-way ANOVA	F (DFn, DFd)	P value
Fig. 1B	Interaction	F (2, 8)=7.874	P =0.0129
	Treatment	F (2, 8)=3.880	P =0.0664
	Week	F (1, 8)=21.53	P =0.0017
	Subject (matching)	F (8, 8)=2.282	P =0.1322
**Tukey's multiple comparisons test**			
Pre	V1 vs V2	V1 vs KLH	V2 vs KLH
**P value**	0.9295	0.3678	0.5545
4 week	V1 vs V2	V1 vs KLH	V2 vs KLH
**P value**	0.0017	0.3894	0.0515

	One-way ANOVA	TC	TG
Fig. 1C	P value	0.0152	0.0210
	Number of groups	3	3
	F	7.398	6.508
**Tukey's multiple comparisons test**		**TC**	**TG**
	V1 vs V2	0.0175	0.0286
	V1 vs KLH	0.9097	0.9953
	V2 vs KLH	0.0470	0.0465
