# Supplementary material for: Development of vaccine for dyslipidemia targeted to a proprotein convertase subtilisin/kexin type 9 (PCSK9) epitope in mice
Source: PLoS One. 2018 Feb 13;13(2):e0191895. doi: 10.1371/journal.pone.0191895 (PMC5811007; doi:10.1371/journal.pone.0191895)
Supplement: S2 Table — (PDF) [file pone.0191895.s010.pdf]

S2 Table. Statistics in Figure 2

	Two-way ANOVA	F (DFn, DFd)	P value
**Fig. 2A**	Interaction	F (16, 120)=9.500	P < 0.0001
	Week	F (8, 120)=28.77	P < 0.0001
	Treatment	F (2, 15)=12.92	P = 0.0005
	Subject (matching)	F (15, 120)=6.261	P < 0.0001
**Tukey's multiple comparisons test**			
Pre	Low vs High	Saline vs Low	Saline vs High
**P value**	ns	ns	ns
2 week	Low vs High	Saline vs Low	Saline vs High
**P value**	ns	ns	ns
4 week	Low vs High	Saline vs Low	Saline vs High
**P value**	ns	ns	ns
6 week	Low vs High	Saline vs Low	Saline vs High
**P value**	ns	P < 0.0001	P < 0.0001
8 week	Low vs High	Saline vs Low	Saline vs High
**P value**	ns	P < 0.01	ns
12 week	Low vs High	Saline vs Low	Saline vs High
**P value**	ns	ns	P < 0.0001
16 week	Low vs High	Saline vs Low	Saline vs High
**P value**	ns	ns	ns
20 week	Low vs High	Saline vs Low	Saline vs High
**P value**	ns	ns	ns
24 week	Low vs High	Saline vs Low	Saline vs High
**P value**	ns	ns	ns

	Two-way ANOVA	F (DFn, DFd)	P value
**Fig. 2B**	Interaction	F (2, 15)=5.390	P = 0.0172
	Treatment	F (2, 15)=3.760	P = 0.0475
	Week	F (1, 15)=18.86	P = 0.0006
	Subject (matching)	F (15, 15)=1.200	P = 0.3641
**Tukey's multiple comparisons test**			
Pre	Low vs High	Low vs Saline	High vs Saline
**P value**	0.9951	0.9625	0.9846
6 week	Low vs High	Low vs Saline	High vs Saline
**P value**	0.2204	0.0271	0.0006

	One-way ANOVA	
**Fig. 2C**	P value	P < 0.0001
	Number of groups	3
	F	227.9
**Tukey's multiple comparisons test**		
	Saline vs Low	P < 0.0001
	Saline vs High	P < 0.0001
	Low vs High	P < 0.05
