# Supplementary material for: Development of vaccine for dyslipidemia targeted to a proprotein convertase subtilisin/kexin type 9 (PCSK9) epitope in mice
Source: PLoS One. 2018 Feb 13;13(2):e0191895. doi: 10.1371/journal.pone.0191895 (PMC5811007; doi:10.1371/journal.pone.0191895)
Supplement: S3 Table — (PDF) [file pone.0191895.s011.pdf]

S3 Table. Statistics in Figure 3A and 3B

	Two-way ANOVA	F (DFn, DFd)	P value
**Fig. 3A**	Interaction	F (4, 30)=12.25	P < 0.0001
	Week	F (2, 15)=3.692	P = 0.0497
	Treatment	F (2, 30)=1.402	P = 0.2618
	Subject (matching)	F (15, 30)=6.145	P < 0.0001
**Tukey's multiple comparisons test**			
Pre	Low vs High	Saline vs Low	Saline vs High
**P value**	0.9868	0.9638	0.9187
6 week	Low vs High	Saline vs Low	Saline vs High
**P value**	0.9971	0.2311	0.2264
24 week	Low vs High	Saline vs Low	Saline vs High
**P value**	0.8354	P < 0.0001	P < 0.0001

	Two-way ANOVA	F (DFn, DFd)	P value
**Fig. 3B LDL**	Interaction	F (4, 30)=1.855	P = 0.1444
	Week	F (2, 15)=0.028	P = 0.9726
	Treatment	F (2, 30)=3.168	P = 0.0565
	Subject (matching)	F (15, 30)=2.417	P = 0.0193
**Tukey's multiple comparisons test**			
Pre	Low vs High	Saline vs Low	Saline vs High
**P value**	0.9955	0.8156	0.7805
6 week	Low vs High	Saline vs Low	Saline vs High
**P value**	0.6412	0.5626	0.1731
24 week	Low vs High	Saline vs Low	Saline vs High
**P value**	0.9403	0.6923	0.5186

	Two-way ANOVA	F (DFn, DFd)	P value
**Fig. 3B VLDL**	Interaction	F (4, 30)=14.22	P < 0.0001
	Week	F (2, 15)=4.895	P = 0.0231
	Treatment	F (2, 30)=8.754	P = 0.0010
	Subject (matching)	F (15, 30)=5.018	P < 0.0001
**Tukey's multiple comparisons test**			
Pre	Low vs High	Saline vs Low	Saline vs High
**P value**	0.9311	0.9242	0.7665
6 week	Low vs High	Saline vs Low	Saline vs High
**P value**	0.8801	0.1815	0.0852
24 week	Low vs High	Saline vs Low	Saline vs High
**P value**	0.8727	P < 0.0001	P < 0.0001
