# Supplementary material for: Development of vaccine for dyslipidemia targeted to a proprotein convertase subtilisin/kexin type 9 (PCSK9) epitope in mice
Source: PLoS One. 2018 Feb 13;13(2):e0191895. doi: 10.1371/journal.pone.0191895 (PMC5811007; doi:10.1371/journal.pone.0191895)
Supplement: S4 Table — (PDF) [file pone.0191895.s012.pdf]

S4 Table. Statistics in Figure 3B and 3C

	Two-way ANOVA	F (DFn, DFd)	P value
**Fig. 3B HDL**	Interaction	F (4, 30)=1.190	P = 0.3355
	Week	F (2, 15)=1.441	P = 0.2676
	Treatment	F (2, 30)=1.316	P = 0.2834
	Subject (matching)	F (15, 30)=12.76	P < 0.0001

Tukey's multiple comparisons test

Pre	Low vs High	Saline vs Low	Saline vs High
**P value**	0.1218	0.9970	0.1458
6 week	Low vs High	Saline vs Low	Saline vs High
**P value**	0.5912	0.9966	0.6884
24 week	Low vs High	Saline vs Low	Saline vs High
**P value**	0.3189	0.8719	0.6682

	Two-way ANOVA	F (DFn, DFd)	P value
**Fig. 3B CM**	Interaction	F (4, 30)=6.481	P = 0.0007
	Week	F (2, 15)=3.073	P = 0.0761
	Treatment	F (2, 30)=17.40	P < 0.0001
	Subject (matching)	F (15, 30)=4.460	P = 0.0002

Tukey's multiple comparisons test

Pre	Low vs High	Saline vs Low	Saline vs High
**P value**	0.9988	0.9133	0.9010
6 week	Low vs High	Saline vs Low	Saline vs High
**P value**	0.6998	0.0395	0.2166
24 week	Low vs High	Saline vs Low	Saline vs High
**P value**	0.8952	0.0022	0.0009

	Two-way ANOVA	F (DFn, DFd)	P value
**Fig. 3C**	Interaction	F (4, 30)=1.129	P = 0.3641
	Week	F (2, 15)=0.6905	P = 0.5166
	Treatment	F (2, 30)=19.24	P < 0.0001
	Subject (matching)	F (15, 30)=3.899	P = 0.0007

Tukey's multiple comparisons test

Pre	Low vs High	Saline vs Low	Saline vs High
**P value**	0.2733	0.5209	0.9264
6 week	Low vs High	Saline vs Low	Saline vs High
**P value**	0.8683	0.6793	0.4138
24 week	Low vs High	Saline vs Low	Saline vs High
**P value**	0.9233	0.8613	0.6716
