# Supplementary material for: Development of vaccine for dyslipidemia targeted to a proprotein convertase subtilisin/kexin type 9 (PCSK9) epitope in mice
Source: PLoS One. 2018 Feb 13;13(2):e0191895. doi: 10.1371/journal.pone.0191895 (PMC5811007; doi:10.1371/journal.pone.0191895)
Supplement: S5 Table — (PDF) [file pone.0191895.s013.pdf]

S5 Table. Statistics in Figure 4

	Two-way ANOVA	F (DFn, DFd)	P value
**Fig. 4B**	Interaction	F (4, 20)=456.5	P < 0.0001
	Treatment	F (4, 20)=3289	P < 0.0001
	cytokine	F (1, 20)=1173	P < 0.0001

Tukey's multiple comparisons test

**IL-4**	**P value**
No-stimulation vs Peptide 2	0.3354
No-stimulation vs Recombinant PCSK9	0.9309
No-stimulation vs KLH	P < 0.0001
No-stimulation vs PHA	P < 0.0001
Peptide 2 vs Recombinant PCSK9	0.7842
Peptide 2 vs KLH	P < 0.0001
Peptide 2 vs PHA	P < 0.0001
Recombinant PCSK9 vs KLH	P < 0.0001
Recombinant PCSK9 vs PHA	P < 0.0001
KLH vs PHA	P < 0.0001
**IFN-gamma**	**P value**
No-stimulation vs Peptide 2	0.9965
No-stimulation vs Recombinant PCSK9	0.9998
No-stimulation vs KLH	P < 0.0001
No-stimulation vs PHA	P < 0.0001
Peptide 2 vs Recombinant PCSK9	0.9998
Peptide 2 vs KLH	P < 0.0001
Peptide 2 vs PHA	P < 0.0001
Recombinant PCSK9 vs KLH	P < 0.0001
Recombinant PCSK9 vs PHA	P < 0.0001
KLH vs PHA	P < 0.0001
