# Supplementary material for: Development of vaccine for dyslipidemia targeted to a proprotein convertase subtilisin/kexin type 9 (PCSK9) epitope in mice
Source: PLoS One. 2018 Feb 13;13(2):e0191895. doi: 10.1371/journal.pone.0191895 (PMC5811007; doi:10.1371/journal.pone.0191895)
Supplement: S6 Table — (PDF) [file pone.0191895.s014.pdf]

S6 Table. Statistics in supplemental-figure 4 and 5

	One-way ANOVA	
S4A-Fig	P value	0.0029
	Number of groups	3
	F	12.04
**Tukey's multiple comparisons test**		
	V1 vs V2	ns
	V1 vs KLH	P < 0.05
	V2 vs KLH	P < 0.01

	Two-way ANOVA	F (DFn, DFd)	P value
S5-Fig	Interaction	F (4, 30)=25.44	P < 0.0001
	Week	F (2, 30)=19.45	P < 0.0001
	Treatment	F (2, 15)=90.42	P < 0.0001
	Subject (matching)	F (15, 30)=2.889	P = 0.0065
**Tukey's multiple comparisons test**			
Low	Pre vs 6 week	Pre vs 8 week	6 week vs 8 week
**P value**	< 0.0001	< 0.0001	< 0.01
High	Pre vs 6 week	Pre vs 8 week	6 week vs 8 week
**P value**	< 0.0001	< 0.01	< 0.0001
Saline	Pre vs 6 week	Pre vs 8 week	6 week vs 8 week
**P value**	ns	ns	ns
