# Supplementary material for: Development of vaccine for dyslipidemia targeted to a proprotein convertase subtilisin/kexin type 9 (PCSK9) epitope in mice
Source: PLoS One. 2018 Feb 13;13(2):e0191895. doi: 10.1371/journal.pone.0191895 (PMC5811007; doi:10.1371/journal.pone.0191895)
Supplement: S7 Table — (PDF) [file pone.0191895.s015.pdf]

S7 Table. Statistics in supplemental-figure 6

	Two-way ANOVA	F (DFn, DFd)	P value
S6B-Fig	Interaction	F (6, 32)=9.240	P < 0.0001
	Vaccine	F (2, 32)=129.4	P < 0.0001
	IgG subclass	F (3, 32)=9.326	P = 0.0001

Tukey's multiple comparisons test		
**V1**		**P value**
	IgG 1 vs IgG	ns
	IgG 2b vs IgG	ns
	IgG 2c vs IgG	ns
	IgG 2b vs IgG 1	ns
	IgG 2c vs IgG 1	ns
	IgG 2c vs IgG 2b	ns
**V2**		**P value**
	IgG 1 vs IgG	ns
	IgG 2b vs IgG	P < 0.01
	IgG 2c vs IgG	P < 0.0001
	IgG 2b vs IgG 1	P < 0.001
	IgG 2c vs IgG 1	P < 0.0001
	IgG 2c vs IgG 2b	ns
**KLH**		**P value**
	IgG 1 vs IgG	ns
	IgG 2b vs IgG	ns
	IgG 2c vs IgG	ns
	IgG 2b vs IgG 1	ns
	IgG 2c vs IgG 1	ns
	IgG 2c vs IgG 2b	ns
