# Supplementary material for: Development of vaccine for dyslipidemia targeted to a proprotein convertase subtilisin/kexin type 9 (PCSK9) epitope in mice
Source: PLoS One. 2018 Feb 13;13(2):e0191895. doi: 10.1371/journal.pone.0191895 (PMC5811007; doi:10.1371/journal.pone.0191895)
Supplement: S8 Table — (PDF) [file pone.0191895.s016.pdf]

S8 Table. Test to analyze the Gaussian distribution for Fig. 2B, 3A, 3B. [Kolmogorov-Smirnov (KS) test]

Fig. 2B	Low pre	High pre	Saline pre	Low 6week	High 6week	Saline 6week
KS normality test						
KS distance	0.2	0.3	0.2	0.2	0.2	0.3
P value	> 0.10	> 0.10	> 0.10	> 0.10	> 0.10	> 0.10
Passed normality test (alpha=0.05)?	Yes	Yes	Yes	Yes	Yes	Yes
P value summary	ns	ns	ns	ns	ns	ns

Fig. 3A	Low pre	High pre	Saline pre	Low 6week	High 6week	Saline 6week	Low 24week	High 24week	Saline 24week
KS normality test									
KS distance	0.1706	0.2639	0.2333	0.1926	0.2093	0.2205	0.2394	0.2735	0.2699
P value	> 0.10	> 0.10	> 0.10	> 0.10	> 0.10	> 0.10	> 0.10	> 0.10	> 0.10
Passed normality test (alpha=0.05)?	Yes	Yes	Yes	Yes	Yes	Yes	Yes	Yes	Yes
P value summary	ns	ns	ns	ns	ns	ns	ns	ns	ns

Fig. 3B VLDL	Low pre	High pre	Saline pre	Low 6week	High 6week	Saline 6week	Low 24week	High 24week	Saline 24week
KS normality test									
KS distance	0.1857	0.2808	0.2189	0.1693	0.2633	0.2185	0.2222	0.3062	0.2535
P value	> 0.10	> 0.10	> 0.10	> 0.10	> 0.10	> 0.10	> 0.10	0.0821	> 0.10
Passed normality test (alpha=0.05)?	Yes	Yes	Yes	Yes	Yes	Yes	Yes	Yes	Yes
P value summary	ns	ns	ns	ns	ns	ns	ns	ns	ns

Fig. 3B CM	Low pre	High pre	Saline pre	Low 6week	High 6week	Saline 6week	Low 24week	High 24week	Saline 24week
KS normality test									
KS distance	0.1878	0.2795	0.2017	0.2448	0.2636	0.2654	0.1819	0.1183	0.2596
P value	> 0.10	> 0.10	> 0.10	> 0.10	> 0.10	> 0.10	> 0.10	> 0.10	> 0.10
Passed normality test (alpha=0.05)?	Yes	Yes	Yes	Yes	Yes	Yes	Yes	Yes	Yes
P value summary	ns	ns	ns	ns	ns	ns	ns	ns	ns
